# Examining psychometric screening scores for depression, anxiety, and posttraumatic stress after fetal KCL injection

**DOI:** 10.1002/pmf2.70109

**Published:** 2025-09-12

**Authors:** Daniel L. Kuhr, Nicola F. Tavella, Alexandra N. Mills, Henri M. Rosenberg, Camila Cabrera, Elizabeth Cochrane, Sara E. Edwards, Allison D. Perelman, Victoria Ly, Sara M. Hachey, Leslie J. Warren, Angela T. Bianco

**Affiliations:** ^1^ Division of Maternal‐Fetal Medicine Icahn School of Medicine at Mount Sinai New York New York USA; ^2^ Division of Maternal‐Fetal Medicine Albert Einstein College of Medicine Bronx New York USA; ^3^ Division of Maternal‐Fetal Medicine Orlando Health Winnie Palmer Hospital for Women and Babies Orlando Florida USA; ^4^ Division of Maternal‐Fetal Medicine Hackensack Meridian School of Medicine Nutley New Jersey USA

**Keywords:** anxiety, depression, posttraumatic stress disorder, PTSD, termination of pregnancy

## Abstract

**Objective:**

To examine the incidence of positive screens for depression, anxiety, and posttraumatic stress among patients undergoing therapeutic abortion at ≥ 22 weeks’ gestation.

**Study Design:**

This was a prospective, observational cohort study of patients undergoing fetal potassium chloride (KCL) injection for induced abortion at ≥ 22 weeks’ gestation. All eligible patients were approached within 48 h of the scheduled termination. After obtaining informed consent, basic demographic information was collected. Patients completed the Edinburgh Postnatal Depression Scale (EPDS), General Anxiety Disorder‐7 (GAD‐7), and Primary Care Post‐Traumatic Stress Disorder‐5 (PC‐PTSD‐5) questionnaires three times, first prior to their KCL procedure and then again 2 and 12 weeks postprocedure. Consents and questionnaires were provided in both English and Spanish. Patients who scored positive on any tool were referred to an in‐house social worker and connected to ongoing mental health care as appropriate.

**Results:**

A total of 53 patients were screened for this study, of whom 30 enrolled. The mean gestational duration at the time of enrollment was 24.7 weeks. Fetal anomaly was the indication for termination in 66.7% of patients. A total of 15 patients (50.0%) completed the 2‐week follow‐up surveys, and 9 patients (30.0%) completed the 12‐week follow‐up surveys. The median (IQR) baseline scores for the EPDS, GAD‐7, and PC‐PTSD‐5 were 13 (11.3), 6.5 (6.5), and 0.5 (2), respectively. The median 2‐week follow‐up scores were 10 (5), 5 (4), and 2 (1), respectively, though these were not statistically significantly different from baseline. The median 12‐week follow‐up scores were 9(3), 4 (3), and 2 (2), respectively, and these were also not statistically significantly different from baseline. There was no difference in the proportion of patients who screened positively on any measures between those patients who completed the follow‐up surveys and those who did not. There were also no differences in the proportion of positive screens by evacuation method or termination indication.

**Conclusions:**

Most patients undergoing termination at ≥ 22 weeks’ gestation at our institution during the study period did so for fetal anomalies. Our results are limited by low return rates for the follow‐up surveys. More research is needed on the long‐term mental health outcomes of patients undergoing termination after 22 weeks.

## 1 INTRODUCTION

Patients seeking termination of pregnancy face multiple psychosocial stressors that can affect their mental health and well‐being. These can include financial, geographical, interpersonal, and time‐sensitive stresses [1]. The geographical and time‐sensitive stresses are likely heightened for patients who need to travel out‐of‐state for care, especially since the *Dobbs v. Jackson* decision in 2022 [2]. Some research has suggested possible association with depression and posttraumatic stress [3] but other studies have not suggested a causal risk, a finding especially present when comparing patients seeking abortion care to those denied to gestational duration limits [4, 5, 6].

The prevalence of depression, anxiety, and posttraumatic stress disorder (PTSD) among pregnant and postpartum people is 14%, 20.7%, and 1.1%, respectively [7, 8]. Among patients terminating for fetal indication, acute depressive symptoms around the time of termination have been reported in a previous cohort, though those symptoms improved with time and professional support [9]. Many different types of resources and support are available to patients, including traditional social work and psychiatric care, local and national support groups, and national hotlines [10, 11, 12].

Evidence regarding mental health outcomes among patients who opt for termination of pregnancy beyond 22 weeks’ gestation is scant, as are studies that examine it over time. Most studies in this population are restricted to patients undergoing termination for fetal indications [13] and only two in a relatively recent systematic review including—but not stratifying by—patients who are terminating for maternal medical reasons [14, 15]. An understanding of patients’ mental health state in this population is critical to improving reproductive clinical care given that many fetal diagnoses do not occur until routine midtrimester ultrasound.

Our institution offers fetal intracardiac potassium chloride (KCL) injection to all patients who undergo a termination of pregnancy at or beyond 22 weeks’ gestation. Furthermore, our institution requires KCL injection for all patients at and beyond 24 weeks’ gestation. Other institutions frequently refer patients to our institution for a fetal KCL injection, with further clinical management occurring at the primary institution. Thus, when designing this study, we determined that centering data collection on the KCL injection was most appropriate, as it is the common experience shared among all patients in this population at our institution.

We sought to study the incidence of positive screens for depression, anxiety, and trauma symptoms in patients prior to their procedures as well in the weeks following them. We hypothesized that the rate of positive screens on the EPDS and GAD‐7 would be elevated (given the acute stress of their situations) but that it would fall over the follow‐up period. We also hypothesized that only a minority of patients would screen positive on the PC‐PTSD‐5 at any point in the study.

## 2 MATERIALS AND METHODS

This was a prospective, observational cohort study of patients undergoing fetal KCL injection for induced abortion at ≥ 22 weeks’ gestation at a single institution from November 1, 2023 through October 31, 2024. All ultrasounds and ultrasound‐guided procedures take place in our outpatient ultrasound unit run by maternal‐fetal medicine. Our institution has multiple social workers who are trained and specialize in perinatal bereavement. Psychometric screening is not a routine part of the outpatient visit for fetal KCL injection at our institution. Patients receiving all of their abortion care at our institution are screened during their intake visit with the division of complex family planning. Patients who underwent termination for fetal anomaly were counseled by maternal‐fetal medicine at their respective home institutions prior to being referred for fetal KCL injection.

All patients aged ≥18 years who were scheduled for KCL injection at ≥ 22 weeks’ gestation were approached within 48 h prior to the scheduled termination. Candidates were identified upon referral to our division for the procedure. Gestational duration was confirmed by review of medical records from our own institution or from the referring institution. Study team members called eligible patients to review the consent and answer any questions. For those wishing to enroll, consent and study data were collected and managed using Research Electronic Data Capture (REDCap) tool hosted at our institution [16, 17]. REDCap is a secure, web‐based software platform designed to support data capture for research studies, providing (1) an intuitive interface for validated data capture; (2) audit trails for tracking data manipulation and export procedures; (3) automated export procedures for seamless data downloads to common statistical packages; and (4) procedures for data integration and interoperability with external sources. They received a copy of the signed document for their records, and study staff documented enrollment internally. For those patients without contact prior to their appointment, study staff reviewed consent in the private examination room, prior to ultrasonography but after procedural consent was completed.

After obtaining informed consent for study participation and their best email address for follow‐up, basic demographic information was abstracted from patients medical records and confirmed with patients. These variables included: age, gestational duration, race/ethnicity, insurance coverage, primary language (English or Spanish), indication for termination (fetal anomaly [genetic or structural], maternal medical indication, or elective), pre‐existing psychiatric diagnoses, and method of uterine evacuation to follow fetal KCL injection. Patients completed the Edinburgh Postnatal Depression Scale (EPDS), General Anxiety Disorder‐7 (GAD‐7), and Primary Care Post‐Traumatic Stress Disorder‐5 (PC‐PTSD‐5) questionnaires up to three times in total. They first completed screening after consent and enrollment, prior to their KCL procedure. Patients were then discharged to their team facilitating the remainder of their termination care with postpartum follow‐up arranged by said team.

All patients were then contacted via secure e‐mail 14 days posttermination and asked to complete the questionnaires a second time electronically. All patients were emailed again 12 weeks posttermination and again asked to complete the three questionnaires. Participants who did not complete the survey within 3 days were emailed again and those who still did not complete it within another 4 days were called once. No voicemails were left for participants who did not answer the phone. Consents and questionnaires were provided in both English and Spanish; only patients fluent in one or both were eligible for enrollment. Positive scores were defined as EPDS ≥ 10, GAD‐7 ≥ 5, and PC‐PTSD‐5 ≥ 3 [18, 19, 20, 21] in order to capture all patients who might benefit from referral to additional mental health care. Patients who scored positively on any tool were referred to an in‐house social worker within 24 h and connected to ongoing mental health care as appropriate. A protocol to send patients to our hospital's Comprehensive Psychiatric Emergency Program (CPEP) was set in the event that a patient reported suicidal ideations.

Baseline characteristics were tabulated. Our primary outcome was the proportion of positive psychometric screening tests at baseline, 2‐week follow‐up, and 12‐week follow‐up. Mann–Whitney *U* tests with a two‐sided alpha of 0.05 were used. Secondary outcomes included the median score and interquartile range (IQR) at each time point. We also performed subgroup analyses of completion of the 2‐ and 12‐week surveys, termination indication (fetal anomaly vs. maternal medical indication or elective), and evacuation method to determine if there were differences between subgroups that could influence results. All data were stored securely in our institutional REDCap server. All analyses were performed using R version 4.4 (April 24, 2024).

Using Cochran's sample size calculation formula (see below), we estimated a 15% incidence of anxiety, depression, and/or PTSD, resulting in a required sample of 25 subjects for 90% power and an alpha of 0.05.

$$n=\frac{z*p\left(1-p\right)}{e^{2}}=\frac{1.96*0.15\left(0.85\right)}{0.01}=25$$

## 3 RESULTS

**FIGURE 1:**
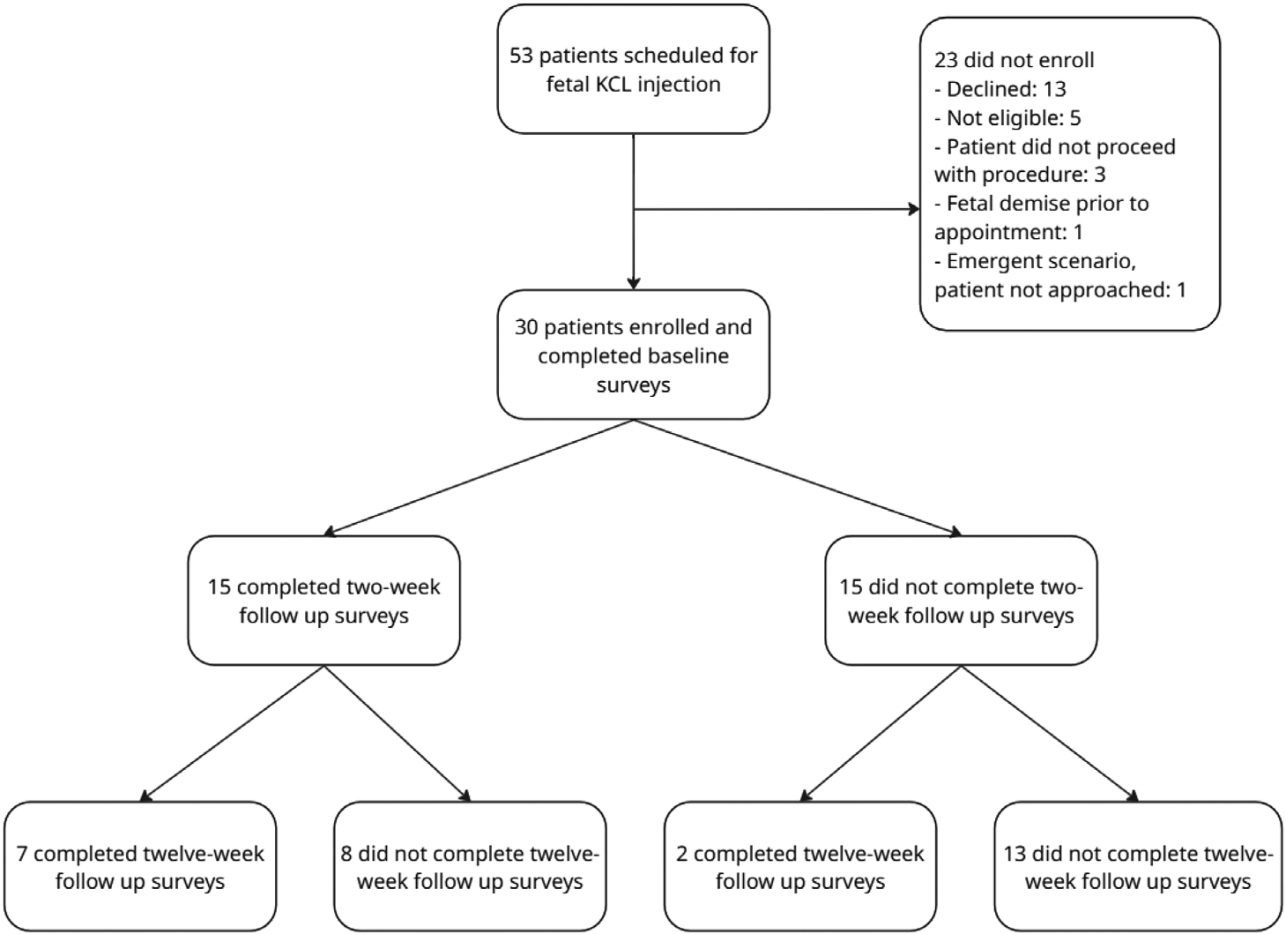
Recruitment and enrollment diagram.

Fifty‐three patients ≥ 18 years old underwent fetal intracardiac KCL injection between November 1, 2023 and October 1, 2024, 30 of whom enrolled in the study and completed the baseline screening tools (Figure 1). Of these 30 patients, 10 (33.3%) were referred from outside New York State, including 4 (13.3%) from outside the New York Metropolitan Region. The average age was 33 years and the mean gestational duration at the time of termination was 24 weeks. Most of the participants were non‐White and the majority were privately insured. Four patients spoke Spanish as their primary language. Two‐thirds of patients’ indication for termination was fetal anomaly. Uterine evacuation following termination was completed by induction of labor in 13 (43%) patients and by dilation and evacuation in the other 17 (57%) patients. Five patients reported pre‐existing anxiety (17%), two reported pre‐existing depression (7%), and one reported diagnosed bipolar disorder (3%) (Table 1).

**TABLE 1:** Demographics.

	Total = 30
Characteristic	*N* (%)
Age at Termination, years (mean ± sd)	33.1 ± 6.4
Gestational Duration at Termination, weeks (mean ± sd)	24.7 ± 1.3
Race and Ethnicity	
White	10 (33.3)
Asian	6 (20.0)
Black/African American	5 (16.7)
Hispanic/Latina	8 (26.7)
Other	1 (3.3)
Insurance Coverage	
Private	19 (63.3)
Public	9 (30.0)
None	2 (6.7)
Primary Language	
English	26 (86.7)
Spanish	4 (13.3)
Patient Referred from Outside New York State	10 (33.3)
Patient Referred from Outside New York City Metro Area	4 (13.3)
Indication for Termination	
Fetal Anomaly (Genetic and/or Structural)	20 (66.7)
Maternal Medical Indication	9 (30.0)
Elective	1 (3.3)
Pre‐existing Psychiatric Diagnoses	
Anxiety	5 (16.7)
Depression	2 (6.7)
Bipolar Disorder	1 (3.3)
Method of Evacuation	
Induction of Labor	13 (43.3)
Dilation and Evacuation	17 (56.7)
Mental Health Resource Uptake	
2‐Week follow‐up (*N* = 15)	
In‐house social work assessment	10 (66.7)
Outside connection to care	3 (30.0)
12‐Week follow‐up (*N* = 9)	
In‐house social work assessment	3 (33.3)
Outside Connection to Care	2 (66.7)

At baseline (*N* = 30), 21 participants (70.0%) scored positive on the EPDS; the median EPDS score (IQR) was 13 (11.3). A total of 19 participants (63.3%) scored positive on the GAD‐7; the median (IQR) was 6.5 (6.5). Seven participants (23.3%) screened positive on the PC‐PTSD‐5; the median (IQR) was 0.5 (2) (Table 2).

**TABLE 2:** Overall psychometric screening scores.

	Baseline		2‐week follow‐up			12‐week follow‐up		
	*N* = 30		*N* = 15			*N* = 9		
Characteristic	Median (IQR)	*N*(%) Scored positive	Median (IQR)	*N*(%) Scored positive	*p* ^*^	Median (IQR)	*N*(%) Scored positive	*p* ^*^
EPDS (score ≥ 10 positive screen)	13 (11.3)	21 (70.0)	10 (5)	10 (66.7)	0.84	9 (3)	3 (33.3)	^a^
GAD7 (score ≥ 5 positive screen)	6.5 (6.5)	19 (63.3)	5 (4)	8 (53.3)	1	4 (3)	4 (44.4)	0.78
PC‐PTSD‐5 (score ≥ 3 positive screen)	0.5 (2)	7 (23.3)	2 (1)	6 (40.0)	0.28	2 (2)	3 (33.3)	1

^a^ All 9 subjects who completed the 12‐week follow‐up scored positively on EPDS at baseline.

* Significance of differences in the proportion of positive scores between baseline and follow‐up.

At 2 weeks (*N* = 15), 10 participants (66.7%) scored positive on the EPDS (*p* = 0.84 when compared to baseline); the median (IQR) was 10 (5). Eight participants (53.3%) scored positive on the GAD‐7 (*p* = 1); the median (IQR) was 5 (4). six participants (40.0%) screened positive on the PC‐PTSD‐5 (*p* = 0.28); the median (IQR) was 2 (1) (Table 2).

At 12 weeks (*N* = 9), 3 participants (33.3%) scored positive on the EPDS; the median (IQR) was 9 (3). Four participants (44.4%) scored positive on the GAD‐7; the median (IQR) was 4 (3). Three participants (33.3%) scored positive on the PC‐PTSD‐5; the median (IQR) was 2 (2). Seven of the participants who completed the 12‐week surveys had also completed the 2‐week surveys and the other two had not.

No patients reported suicidal ideations at any point in the study and thus none were referred to our hospital's CPEP.

In subgroup analyses, there were no differences in the rates of positive screens between the patients who did and did not complete both the 2‐ and 12‐week screens (Table 3). There was also no difference in the baseline rate of positive screens on any measure when stratifying by termination indication and posttermination uterine evacuation method (Table 4).

**TABLE 3:** Psychometric screening scores stratified by follow‐up completion.

	Completed 2‐week follow‐up			Completed 12‐week follow‐up		
	Yes, *N* = 15	No, *N* = 15		Yes, *N* = 9	No, *N* = 21	
Characteristic	*N* (%)	*N* (%)	*p*	*N* (%)	*N* (%)	*p*
EPDS positive screen at baseline	11 (73.3)	10 (66.7)	1	9 (100.0)	12 (57.1)	0.06
GAD7 positive screen at baseline	10 (66.7)	9 (60.0)	1	7 (77.8)	12 (57.1)	0.51
PC‐PTSD‐5 positive screen at baseline	2 (13.3)	5 (33.3)	0.39	3 (33.3)	4 (19.0)	0.71

**TABLE 4:** Psychometric screening scores stratified by termination characteristics.

	Termination for fetal anomaly			Evacuation method		
	Yes, *N* = 20	No, *N* = 10		Induction of labor, *N* = 13	Dilation and evacuation, *N* = 17	
Characteristic	*N*(%)	*N*(%)	*p*	*N*(%)	*N*(%)	*p*
EPDS positive screen at baseline	16 (80.0)	5 (50.0)	0.2	11 (84.6)	10 (58.8)	0.26
GAD7 positive screen at baseline	15 (75.0)	4 (40.0)	0.14	11 (84.6)	8 (47.1)	0.08
PC‐PTSD‐5 positive screen at baseline	5 (25.0)	2 (20.0)	1	2 (15.4)	5 (29.4)	0.64

## 4 DISCUSSION

To our knowledge, this is the largest cohort of patients undergoing pregnancy termination at ≥ 22 weeks’ gestation for whom psychometric screening has been measured prior to the procedure as well as postprocedure sequentially. We found that over half of patients screened positive for depression (EPDS ≥ 10) and/or anxiety (GAD‐7 ≥ 5) at the time of the scheduled termination. As time passed, the proportion of patients who screened positive fell, though these differences were not statistically significant. There was a lower rate of patients screening positive for PTSD prior to their procedures, which appeared stable as time progressed.

A high incidence of positive screens for posttraumatic stress (24%), moderate‐to‐severe anxiety (24%), and moderate‐to‐severe depression (11%) has been reported 1 month following early pregnancy loss, which subsequently decreased but remained elevated at 18%, 17%, and 6%, respectively [22]. With regard to abortion specifically, a 2013 systemic review reported mixed results on the association between termination and anxiety, depression, and trauma [3]. Notably, the authors’ ability to comment on a correlation between abortion and subsequent risks for mental health was limited due to heterogeneity of patient populations and tools used by various studies. The Turnaway Study has evaluated the mental health outcomes of patients seeking abortion care who were and were not able to receive the care they sought and has consistently found no difference in the rates of mental health diagnoses or changes in self‐esteem between the two groups, suggesting that abortion itself does not cause adverse mental health outcomes [4, 5, 23, 24].

Specifically regarding termination in the late second and early third trimester, the evidence for its link with mental health outcomes is sparse. Most relevant to our population, Kerns et al. reported that among patients who undergo abortion for fetal anomalies, abortion stigma and higher self‐judgment are associated with posttermination grief but were not significantly associated with posttraumatic stress or other mental health outcomes [25]. Given that fetal anomaly was the most common indication for termination in our population, this contextualizes the varied experiences patients face after termination.

Our results should *not* be interpreted to suggest a causative relationship between abortion care and mental health disorders. Psychometric screening scores are not equivalent to formal diagnosis with DSM‐5 criteria, and even patients who have severe scores require further evaluation by a trained and licensed mental health professional. Rather, our results suggest that the patients who undergo termination ≥ 22 weeks’ gestation are more likely to do so due to a medical indication and are under greater psychological stress. These patients should be given ample resources to aid them throughout and after their abortion process. For example, our institution offers a support group for patients who undergo medically indicated termination of pregnancy, to which many of our enrolled patients were referred. Regional and national resources also exist for institutions that do not have their own similar support group.

Our study's biggest strength is that it is the first prospective psychometric cohort study of patients undergoing termination of pregnancy during and after the periviability period. Our use of the EPDS, GAD‐7, and PC‐PTSD‐5 increases the applicability of our findings, as they are easy to use and among the tools recommended by the American College of Obstetricians and Gynecologists for psychometric screening [18]. Additionally, the EPDS and GAD‐7 can potentially capture the patients’ mental health burden even in cases without a formal psychiatric diagnosis [26, 27]. Our patients, though largely native to our home state, included patients from multiple states, broadening the generalizability. Our study is limited by its small sample size and limited response to follow‐up questionnaires. However, given how rare termination is at this stage of pregnancy, the small sample size is not surprising. The high attrition rate could potentially bias our results if the patients who failed to follow up would have had relatively higher or lower screening scores at 2 and 12 weeks. Also, by restricting to only English‐ and Spanish‐speaking patients, there is a potential for bias in excluding patients who speak other languages and come from other cultures, thus limiting our generalizability.

Facilities that provide abortion services should systematically support patients undergoing termination, particularly during or beyond periviability. Given the high mental health burden demonstrated in this cohort, we suspect that most patients with similar circumstances would benefit from both screening and mental health resources. Additionally, screening should be incorporated into these patients’ postpartum and postoperative visits as is the case for postpartum visits following live births [28], with a low threshold to provide patients with additional resources. Future research should expand on this and follow a larger cohort and consider additional psychometric tests to assess well‐being over time. Large cohorts may allow for imputation analysis to account for missing responses in follow‐up, though our small sample size precludes that here without introducing new bias. Finally, scientists and clinicians studying this topic should develop robust protocols to improve follow‐up rates in future study designs while still being respectful of patients’ privacy.

## 5 CONCLUSION

In our cohort of 30 patients undergoing fetal KCL injection for termination ≥ 22 weeks’ gestation, there was a high incidence of positive screens for anxiety, depression, and PTSD symptoms using validated psychometric tools. Clinicians should routinely psychometrically screen these patients and offer referrals as appropriate.

## CONFLICT OF INTEREST STATEMENT

The authors declare no conflicts of interest.

## FUNDING INFORMATION

No external funding supported this work.

## ETHICS STATEMENT

This study was approved by the Institutional Review Board at the Icahn School of Medicine at Mount Sinai. All participants granted consent prior to participation.

## Data Availability

Data are not publicly available given the sensitive nature of the data, but can be provided upon reasonable request to the authors.

## References

[1] Odum, T. , O. Heymann , A. N. Turner , K. Rivlin , and D. Bessett . 2023. “Assessing Psychosocial Costs: Ohio Patients' Experiences Seeking Abortion Care.” Contraception 117: 45–9. 36087646 10.1016/j.contraception.2022.08.007

[2] Roussos‐Ross, K. , M. D. Hansen , and A. Monaco . 2025. “Post‐Traumatic Stress Disorder and the Mental Burden Resulting from the Dobbs Decision.” Obstetrics and Gynecology Clinics of North America 52(1): 81–91. 39880568 10.1016/j.ogc.2024.09.001

[3] Bellieni, C. V. , and G. Buonocore . 2013. “Abortion and Subsequent Mental Health: Review of the Literature.” Psychiatry and Clinical Neurosciences 67(5): 301–10. 23859662 10.1111/pcn.12067

[4] Foster, D. G. , J. R. Steinberg , S. C. M. Roberts , J. Neuhaus , and M. A. Biggs . 2015. “A Comparison of Depression and Anxiety Symptom Trajectories between Women Who Had an Abortion and Women Denied One.” Psychological Medicine 45(10): 2073–82. 25628123 10.1017/S0033291714003213 PMC5004731

[5] Biggs, M. A. , J. M. Neuhaus , and D. G. Foster . 2015. “Mental Health Diagnoses 3 Years after Receiving or Being Denied an Abortion in the United States.” American Journal of Public Health 105(12): 2557–63. 26469674 10.2105/AJPH.2015.302803 PMC4638270

[6] Munk‐Olsen, T. , T. M. Laursen , C. B. Pedersen , Ø. Lidegaard , and P. B. Mortensen . 2011. “Induced First‐Trimester Abortion and Risk of Mental Disorder.” New England Journal of Medicine 364(4): 332–9. 21268725 10.1056/NEJMoa0905882

[7] Wisner, K. L. , D. K. Y. Sit , M. C. McShea , D. M. Rizzo , R. A. Zoretich , C. L. Hughes , H. F. Eng , et al. 2013. “Onset Timing, Thoughts of Self‐Harm, and Diagnoses in Postpartum Women With Screen‐Positive Depression Findings.” JAMA Psychiatry 70(5): 490–8. 23487258 10.1001/jamapsychiatry.2013.87 PMC4440326

[8] Fawcett, E. J. , N. Fairbrother , M. L. Cox , I. R. White , and J. M. Fawcett . 2019. “The Prevalence of Anxiety Disorders During Pregnancy and the Postpartum Period: A Multivariate Bayesian Meta‐Analysis.” Journal of Clinical Psychiatry 80(4): 18r12527. 10.4088/JCP.18r12527 PMC6839961 31347796

[9] di Giacomo, E. , R. Pessina , V. Placenti , F. Pescatore , F. Colmegna , and M. Clerici . 2022. “Therapeutic Termination of Pregnancy and Major Psychiatric Outcomes in Parents.” Psychiatry Research 317: 114779. 36030698 10.1016/j.psychres.2022.114779

[10] Hope After Loss . Support Groups. [cited August 6, 2025]. https://www.hopeafterloss.org/support‐groups.

[11] Planned Parenthood of Orange & San Bernadino Countines, Inc . Support After Miscarriage or Abortion. [cited August 6, 2025]. https://www.plannedparenthood.org/planned‐parenthood‐orange‐san‐bernardino/patients/miscarriage‐support.

[12] National Abortion Federation . National Abortion Hotline. [cited August 6, 2025]. https://prochoice.org/patients/naf‐hotline/.

[13] González‐Ramos, Z. , E. Zuriguel‐Pérez , N. Albacar‐Riobóo , and L. Casadó‐Marín . 2021. “The Emotional Responses of Women When Terminating a Pregnancy for Medical Reasons: A Scoping Review.” Midwifery 103: 103095. 34320417 10.1016/j.midw.2021.103095

[14] Aktürk, Ü. , and B. Erci . 2019. “The Effect of Watson's Care Model on Anxiety, Depression, and Stress in Turkish Women.” Nursing Science Quarterly 32(2): 127–34. 30888300 10.1177/0894318419826257

[15] Aghakhani, N. , M. Cleary , A. Zarei , and V. Lopez . 2018. “Women's Attitudes to Safe‐Induced Abortion in Iran: Findings from a Pilot Survey.” Journal of Advanced Nursing 74(1): 61–4. 28726247 10.1111/jan.13393

[16] Harris, P. A. , R. Taylor , B. L. Minor , V. Elliott , M. Fernandez , L. O'Neal , L. McLeod , et al. 2019. “The REDCap Consortium: Building an International Community of Software Platform Partners.” Journal of Biomedical Informatics 95: 103208. 31078660 10.1016/j.jbi.2019.103208 PMC7254481

[17] Harris, P. A. , R. Taylor , R. Thielke , J. Payne , N. Gonzalez , and J. G. Conde . 2009. “Research Electronic Data Capture (REDCap)—A Metadata‐Driven Methodology and Workflow Process for Providing Translational Research Informatics Support.” Journal of Biomedical Informatics 42(2): 377–81. 18929686 10.1016/j.jbi.2008.08.010 PMC2700030

[18] 2023. “Screening and Diagnosis of Mental Health Conditions During Pregnancy and Postpartum: ACOG Clinical Practice Guideline No. 4.” Obstetrics & Gynecology 141(6): 1232–61. 37486660 10.1097/AOG.0000000000005200

[19] Cox, J. 1996. “Validation of the Edinburgh Postnatal Depression Scale (EPDS) in Non‐Postnatal Women.” Journal of Affective Disorders 39(3): 185–9. 8856422 10.1016/0165-0327(96)00008-0

[20] Bovin, M. J. , R. Kimerling , F. W. Weathers , A. Prins , B. P. Marx , E. P. Post , and P. P. Schnurr , 2021. “Diagnostic Accuracy and Acceptability of the Primary Care Posttraumatic Stress Disorder Screen for the Diagnostic and Statistical Manual of Mental Disorders (Fifth Edition) Among US Veterans.” JAMA Network Open 4(2): e2036733. 33538826 10.1001/jamanetworkopen.2020.36733 PMC7862990

[21] 2007. “Anxiety Disorders in Primary Care: Prevalence, Impairment, Comorbidity, and Detection.” Annals of Internal Medicine 146(5): 317–25. 17339617 10.7326/0003-4819-146-5-200703060-00004

[22] Farren, J. , M. Jalmbrant , N. Falconieri , N. Mitchell‐Jones , S. Bobdiwala , M. Al‐Memar , S. Tapp , et al. 2020. “Posttraumatic Stress, Anxiety and Depression Following Miscarriage and Ectopic Pregnancy: A Multicenter, Prospective, Cohort Study.” American Journal of Obstetrics and Gynecology 222(4): 367.e1–22. 10.1016/j.ajog.2019.10.102 31953115

[23] Biggs, M. A. , B. Rowland , C. E. McCulloch , and D. G. Foster . 2016. “Does Abortion Increase Women's Risk for Post‐Traumatic Stress? Findings from a Prospective Longitudinal Cohort Study.” BMJ Open 6(2): e009698. 10.1136/bmjopen-2015-009698 PMC4746441 26832431

[24] Biggs, M. A. , U. D. Upadhyay , J. R. Steinberg , and D. G. Foster . 2014. “Does Abortion Reduce Self‐Esteem and Life Satisfaction?.” Quality of Life Research 23(9): 2505–13. 24740325 10.1007/s11136-014-0687-7 PMC4186981

[25] Kerns, J. , M. Cheeks , A. Cassidy , G. Pearlson , and B. Mengesha . 2022. “Abortion Stigma and Its Relationship with Grief, Post‐traumatic Stress, and Mental Health‐Related Quality of Life after Abortion for Fetal Anomalies.” Women's Health Reports 3(1): 385–94. 10.1089/whr.2021.0027 PMC8994429 35415714

[26] McCabe‐Beane, J. E. , L. S. Segre , Y. Perkhounkova , S. Stuart , and M. W. O'Hara . 2016. “The Identification of Severity Ranges for the Edinburgh Postnatal Depression Scale.” Journal of Reproductive and Infant Psychology 34(3): 293–303.

[27] Spitzer, R. L. , K. Kroenke , J. B. W. Williams , and B. Löwe . 2006. “A Brief Measure for Assessing Generalized Anxiety Disorder: The GAD‐7.” Archives of Internal Medicine 166(10): 1092–7. 16717171 10.1001/archinte.166.10.1092

[28] 2018. “ACOG Committee Opinion No. 736: Optimizing Postpartum Care.” Obstetrics & Gynecology 131(5): e140–e50. 29683911 10.1097/AOG.0000000000002633

